# MacFrag: segmenting large-scale molecules to obtain diverse fragments with high qualities

**DOI:** 10.1093/bioinformatics/btad012

**Published:** 2023-01-13

**Authors:** Yanyan Diao, Feng Hu, Zihao Shen, Honglin Li

**Affiliations:** Shanghai Key Laboratory of New Drug Design, School of Pharmacy, East China University of Science and Technology, Shanghai 200237, China; Shanghai Key Laboratory of New Drug Design, School of Pharmacy, East China University of Science and Technology, Shanghai 200237, China; Shanghai Key Laboratory of New Drug Design, School of Pharmacy, East China University of Science and Technology, Shanghai 200237, China; Shanghai Key Laboratory of New Drug Design, School of Pharmacy, East China University of Science and Technology, Shanghai 200237, China; Innovation Center for AI and Drug Discovery, East China Normal University, Shanghai 200062, China; Lingang Laboratory, Shanghai 200031, China

## Abstract

**Summary:**

Construction of high-quality fragment libraries by segmenting organic compounds is an important part of the drug discovery paradigm. This article presents a new method, MacFrag, for efficient molecule fragmentation. MacFrag utilized a modified version of BRICS rules to break chemical bonds and introduced an efficient subgraphs extraction algorithm for rapid enumeration of the fragment space. The evaluation results with ChEMBL dataset exhibited that MacFrag was overall faster than BRICS implemented in RDKit and modified molBLOCKS. Meanwhile, the fragments acquired through MacFrag were more compliant with the ‘Rule of Three’.

**Availability and implementation:**

https://github.com/yydiao1025/MacFrag.

**Supplementary information:**

Supplementary data are available at *Bioinformatics* online.

## 1 Introduction

Decomposing molecules into small pieces provides valuable ways to construct fragment libraries with diversity and chemical feasibility, which are critical for successful fragment-based *de novo* drug design and scaffold hopping by fragment replacement (Erlanson *et al.*, 2019; Hu *et al.*, 2017). RECAP (Lewell *et al.*, 1998) and BRICS (Degen *et al.*, 2008) algorithms define series of cleavage bonds based on retrosynthetic chemistry and are widely used as ground rules in other practical fragmentation tools, such as molBLOCKS (Ghersi and Singh, 2014) and *e*MolFrag (Liu *et al.*, 2017), featured with extended functions. Alternative non-retrosynthetic bond breaking rules, although relatively rare, have also been reported (Firth *et al.*, 2015; Vainio *et al.*, 2013).

These cleavage rules available typically break acyclic bonds in order to keep ring moieties intact. The restriction has its rationality under the circumstances of small ring systems. However, the number of macrocycles has increased considerably over the past decade (Diaz *et al.*, 2021; Whitty *et al.*, 2016), due to the growing researches focused on them as drug candidates. Therefore, excluding cyclic bonds may be detrimental to improving fragment diversity and hinder access to distinctive fragments, especially those likely to favor macrocyclization of linear compounds (Cummings and Sekharan, 2019). Besides, most of the fragmentation programs available tend to cut molecules into the smallest building blocks, which might cause the disruption of certain pharmacophores, e.g. ester and amide groups. BRICS algorithm implemented in RDKit (http://www.rdkit.org) offers an extra option to output all intermediate substructures of a given molecule, aiming to thoroughly sample the fragment space. Nevertheless, for those chemical entities with high-molecular weights and plenty of breaking bonds, this would result in exponential increase of runtime and redundant large substructures that are not suited as fragments. A modified molBLOCKS program (Heikamp *et al.*, 2018) was designed for exhaustive fragmentation purpose through combining the smallest building blocks to large subgroups. Although this program is capable of controlling the sizes of output fragments by specifying the maximum number of building blocks they contain, its computational performance on large datasets has not been stated in the study.

**Fig. 1.**
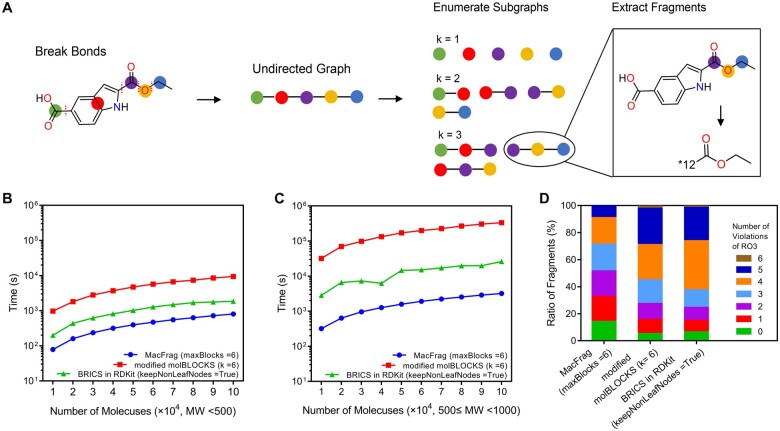
Workflow and performance of MacFrag. (**A**) Overview of MacFrag. ‘12’ is the SMARTS index of the atom that was removed. The computational performances of MacFrag, modified molBLOCKS, and BRICS implemented in RDKit using molecules randomly selected from ChEMBL database with molecular weight (MW) lower than 500 (**B**) and ranging from 500 to 1000 (**C**), respectively. Each resulted running time is the average value of three independent experiments. (**D**) Evaluation of qualities of fragments obtained by the three programs.

Herein, we proposed the fragmentation approach MacFrag (Fig. 1A), on account of solving the issues mentioned above simultaneously. The main functions and innovations of MacFrag are summarized as follows:

- Extend the BRICS rules and take cyclic breaking bonds into account so as to increase the probability of acquiring novel fragments.
- Construct fragments with different numbers of building blocks, in order to cover a wider fragment space.
- Take the molecule as a graph of building block nodes, and introduce the bounded connected induced subgraphs enumeration algorithm named Simple (Komusiewicz and Sommer, 2021; Wernicke, 2006) to speed up the segmentation process.

## 2 Implementation

### 2.1 Cleavage bonds recognition

Given a molecule, the first step of MacFrag is to recognize all cleavage bonds and cut the molecule into the smallest building blocks. We referred to the BRICS rules implemented in RDKit and deleted the restriction to acyclic bonds. Eventually, 19 atomic environments were defined using SMARTS strings (Supplementary Table S1), which were subsequently combined into 49 bonds to be cleaved (Supplementary Table S2). We set a user-definable parameter maxSR, and the ring structure containing the number of atoms equal to or less than this value will remain intact. A very large value of maxSR means that the cyclic bonds will not be split, which is the same as the original BRICS version. It is also possible for users to customize the fragmentation rules according to the source codes available at https://github.com/yydiao1025/MacFrag.

### 2.2 Subgraphs and fragments enumeration

The molecule is simplified as an undirected graph by treating the building blocks as nodes and the chemical bonds that connect them as edges. The efficient algorithm Simple was applied for enumerating all connected induced subgraphs with the specified maximum number of nodes. Instead of combining building blocks, the subgraphs were mapped to the original molecules and the fragments were extracted after removing extra atoms and bonds. The output fragments will be labeled with dummy symbols to specify the position of breaking bonds, and redundant fragments of the same molecule will be filtered. A parameter maxBlocks was defined to control the maximum number of building blocks that the fragments contain.

## 3 Results

The computational performance of MacFrag was evaluated and compared to the other two methods, BRICS in RDKit and modified molBLOCKS, which also could execute fragment enumerations. RECAP rules were used in modified molBLOCKS to specify the bonds to break. All simulations were performed on a Linux server equipped with two 2.4 GHz 12-core Intel Xeon CPUs, 256 GB RAM and 2 TB SSD primary storage. Large amount of molecule datasets with the numbers ranging from 10 000 to 100 000 was randomly selected from ChEMBL database (Mendez *et al.*, 2019), and each resulted running time is the average value of three independent experiments. The results showed that MacFrag had uniformly better computational performance (Fig. 1B and C). For small molecules with molecular weights lower than 500, MacFrag is approximately 2.5- and 11.8-fold faster than BRICS and modified molBLOCKS, respectively. As molecular weights increase to 500–1000, the superiority of MacFrag in terms of running speed is more significant, which is approximately and 7.9- and 104-fold faster than BRICS and modified molBLOCKS, respectively.

Subsequently, segmentation of large-scale molecules was performed to evaluate the qualities of fragments obtained by the three programs. We collected 1 921 745 molecules with molecular weights lower than 1000 from ChEMBL database and 10 336 743, 20 733 058 and 27 468 335 unique fragments were yielded by MacFrag, BRICS, and modified molBLOCKS, respectively. The properties in terms of the ‘Rule of Three’ (RO3) (Jhoti *et al.*, 2013), were calculated using RDKit and plotted in Supplementary Figure S1. Despite highly significant differences (*P *< 0.0001) were noted for each property of fragments generated by two different fragmentation tools, the properties of MacFrag fragments are distributed in lower regions. Take molecular weight for example, the fragments of MacFrag have a median value of 288.3, whereas the medians of modified molBLOCKS and BRICS are 369.2 and 389.4, respectively. Among these fragments, about 13% (1 360 909) of MacFrag can pass through the RO3, while only 5% (1 381 220) and 6% (1 297 338) for modified molBLOCKS and BRICS, respectively. Furthermore, as the maximum numbers of violations of RO3 increase, the ratios of fragments obtained by MacFrag remain higher (Fig. 1D). Among the RO3-compliant fragments, 82 758 are peculiar to MacFrag, which should be largely attributed to cleavage of cyclic bonds (examples shown in Supplementary Fig. S2).

## 4 Conclusion

The MacFrag method proposed in this study could enumerate the fragment space of molecules in a highly efficient manner, implying its potential for the segmentation of large-scale database, especially those containing large molecules. Furthermore, the modified BRICS rules facilitate access to novel fragments while maintaining chemical feasibility. It is expected that MacFrag will serve as a useful tool for the construction of high-quality fragment libraries.

## Supplementary Material

btad012_Supplementary_Data

## Data Availability

The data underlying this article are available in https://github.com/yydiao1025/MacFrag.
