# Supplementary material for: MacFrag: segmenting large-scale molecules to obtain diverse fragments with high qualities
Source: Bioinformatics. 2023 Jan 13;39(1):btad012. doi: 10.1093/bioinformatics/btad012 (PMC9872447; doi:10.1093/bioinformatics/btad012)
Supplement: btad012_Supplementary_Data [file btad012_supplementary_data.docx]

**MacFrag: segmenting large-scale molecules to obtain diverse fragments with high qualities**

Yanyan Diao^1^, Feng Hu^1^, Zihao Shen^1^, Honglin Li^1,2,3,*^

^1^Shanghai Key Laboratory of New Drug Design, School of Pharmacy, East China University of Science and Technology, Shanghai 200237, China, ^2^ Innovation Center for AI and Drug Discovery, East China Normal University, Shanghai 200062, China, ^3^ Lingang Laboratory, Shanghai 200031, ChinaHNJIUK

*To whom correspondence should be addressed.

**Table S1.** SMARTS representations of the 19 atomic environments for molecule fragmentation.

**Table S2.** SMARTS representations of the 49 chemical bonds to be cleaved in MacFrag.

**Fig. S1.** Property values of fragments generated by MacFrag (maxBlocks =6), modified molBLOCKS (k =6), and BRICS implemented in RDKit (keepNonLeafNoldes =True), repsectively. The properties relevant to the RO3 include molecular weight （MW, <300）, the number of hydrogen bond donors (NHD, ≤3), the number of hydrogen bond acceptors (NHA, ≤3), logP ( ≤3), the number of rotatable bonds (NROT, ≤3), and polar surface area (PSA, ≤60). The two-sided Wilcoxon rank-sum test was used to calculate the statistical difference of each property and p value depicted by ****p < 0.0001. The median values are labeled to further display the discrepancies between the groups.

**Fig. S2.** Distinctive RO3-compliant fragments generated by MacFrag by breaking cyclic bonds. All breaking bonds identified by MacFrag are labeled with red dash lines. These fragments couldn’t be acquired by either BRICS or modified molBLOCKS through segmenting ChEMBL compounds with molecular weights lower than 1000.

**Table S1.** SMARTS representations of the 19 atomic environments for molecule fragmentation.

| index | SMARTS |
| --- | --- |
| L1 | [C;D3]([#0,#6,#7,#8])(=O) |
| L2 | [O;D2]-[#0,#6,#1] |
| L3 | [C;!D1;!$(C=*)]-[#6] |
| L4 | [N;!D1;!$(N=*);!$(N-[!#6;!#16;!#0;!#1]);!$([N;R]@[C;R]=O)] |
| L5 | [C;D2,D3]-[#6] |
| L6 | [C;!D1;!$(C!-*)] |
| L61 | [C;R1;!D1;!$(C!-*)] |
| L7 | [n;+0;$(n(:[c,n,o,s]):[c,n,o,s])] |
| L8 | [N;R;$(N(@C(=O))@[#6,#7,#8,#16])] |
| L9 | [S;D2](-[#0,#6]) |
| L10 | [S;D4]([#6,#0])(=O)(=O) |
| L11 | [C;$(C(-;@[C,N,O,S])-;@[N,O,S])] |
| L111 | [C;R2;$(C(-;@[C,N,O,S])-;@[N,O,S])] |
| L112 | [C;R1;$(C(-;@[C,N,O,S;R2])-;@[N,O,S;R2])] |
| L12 | [c;$(c(:[c,n,o,s]):[n,o,s])] |
| L13 | [C;$(C(-;@C)-;@C)] |
| L131 | [C;R2;$(C(-;@C)-;@C)] |
| L132 | [C;R1;$(C(-;@[C;R2])-;@[C;R2])] |
| L14 | [c;$(c(:c):c)] |

**Table S2.** SMARTS representations of the 49 chemical bonds to be cleaved in MacFrag.

| No. | Breaking bonds |
| --- | --- |
| 1 | [$([C;D3]([#0,#6,#7,#8])(=O))]-[$([O;D2]-[#0,#6,#1])] |
| 2 | [$([C;D3]([#0,#6,#7,#8])(=O))]-[$([N;!D1;!$(N=*);!$(N-[!#6;!#16;!#0;!#1]);!$([N;R]@[C;R]=O)])] |
| 3 | [$([C;D3]([#0,#6,#7,#8])(=O))]-[$([N;R;$(N(@C(=O))@[#6,#7,#8,#16])])] |
| 4 | [$([C;D3]([#0,#6,#7,#8])(=O))]-[$([C;$(C(-;@[C,N,O,S])-;@[N,O,S])])] |
| 5 | [$([C;D3]([#0,#6,#7,#8])(=O))]-[$([c;$(c(:[c,n,o,s]):[n,o,s])])] |
| 6 | [$([C;D3]([#0,#6,#7,#8])(=O))]-[$([C;$(C(-;@C)-;@C)])] |
| 7 | [$([C;D3]([#0,#6,#7,#8])(=O))]-[$([c;$(c(:c):c)])] |
| 8 | [$([O;D2]-[#0,#6,#1])]-[$([C;!D1;!$(C=*)]-[#6])] |
| 9 | [$([O;D2]-[#0,#6,#1])]-[$([C;$(C(-;@[C,N,O,S])-;@[N,O,S])])] |
| 10 | [$([O;D2]-[#0,#6,#1])]-[$([c;$(c(:[c,n,o,s]):[n,o,s])])] |
| 11 | [$([O;D2]-[#0,#6,#1])]-[$([C;$(C(-;@C)-;@C)])] |
| 12 | [$([O;D2]-[#0,#6,#1])]-[$([c;$(c(:c):c)])] |
| 13 | [$([C;!D1;!$(C=*)]-[#6])]-[$([N;!D1;!$(N=*);!$(N-[!#6;!#16;!#0;!#1]);!$([N;R]@[C;R]=O)])] |
| 14 | [$([C;!D1;!$(C=*)]-[#6])]-[$([S;D2](-[#0,#6]))] |
| 15 | [$([N;!D1;!$(N=*);!$(N-[!#6;!#16;!#0;!#1]);!$([N;R]@[C;R]=O)])]-[$([S;D4]([#6,#0])(=O)(=O))] |
| 16 | [$([N;!D1;!$(N=*);!$(N-[!#6;!#16;!#0;!#1]);!$([N;R]@[C;R]=O)])]-[$([c;$(c(:[c,n,o,s]):[n,o,s])])] |
| 17 | [$([N;!D1;!$(N=*);!$(N-[!#6;!#16;!#0;!#1]);!$([N;R]@[C;R]=O)])]-[$([c;$(c(:c):c)])] |
| 18 | [$([N;!D1;!$(N=*);!$(N-[!#6;!#16;!#0;!#1]);!$([N;R]@[C;R]=O)])]-[$([C;$(C(-;@[C,N,O,S])-;@[N,O,S])])] |
| 19 | [$([N;!D1;!$(N=*);!$(N-[!#6;!#16;!#0;!#1]);!$([N;R]@[C;R]=O)])]-[$([C;$(C(-;@C)-;@C)])] |
| 20 | [$([C;D2,D3]-[#6])]=[$([C;D2,D3]-[#6])] |
| 21 | [$([C;!D1;!$(C!-*)])]-[$([n;+0;$(n(:[c,n,o,s]):[c,n,o,s])])] |
| 22 | [$([C;!D1;!$(C!-*)])]-[$([N;R;$(N(@C(=O))@[#6,#7,#8,#16])])] |
| 23 | [$([C;!D1;!$(C!-*)])]-;!@[$([C;$(C(-;@[C,N,O,S])-;@[N,O,S])])] |
| 24 | [$([C;!D1;!$(C!-*)])]-[$([c;$(c(:[c,n,o,s]):[n,o,s])])] |
| 25 | [$([C;!D1;!$(C!-*)])]-;!@[$([C;$(C(-;@C)-;@C)])] |
| 26 | [$([C;!D1;!$(C!-*)])]-[$([c;$(c(:c):c)])] |
| 27 | [$([C;R1;!D1;!$(C!-*)])]-;@[$([C;R2;$(C(-;@[C,N,O,S])-;@[N,O,S])])] |
| 28 | [$([C;R1;!D1;!$(C!-*)])]-;@[$([C;R2;$(C(-;@C)-;@C)])] |
| 29 | [$([n;+0;$(n(:[c,n,o,s]):[c,n,o,s])])]-[$([C;$(C(-;@[C,N,O,S])-;@[N,O,S])])] |
| 30 | [$([n;+0;$(n(:[c,n,o,s]):[c,n,o,s])])]-[$([c;$(c(:[c,n,o,s]):[n,o,s])])] |
| 31 | [$([n;+0;$(n(:[c,n,o,s]):[c,n,o,s])])]-[$([C;$(C(-;@C)-;@C)])] |
| 32 | [$([n;+0;$(n(:[c,n,o,s]):[c,n,o,s])])]-[$([c;$(c(:c):c)])] |
| 33 | [$([N;R;$(N(@C(=O))@[#6,#7,#8,#16])])]-[$([C;$(C(-;@[C,N,O,S])-;@[N,O,S])])] |
| 34 | [$([N;R;$(N(@C(=O))@[#6,#7,#8,#16])])]-[$([c;$(c(:[c,n,o,s]):[n,o,s])])] |
| 35 | [$([N;R;$(N(@C(=O))@[#6,#7,#8,#16])])]-[$([C;$(C(-;@C)-;@C)])] |
| 36 | [$([N;R;$(N(@C(=O))@[#6,#7,#8,#16])])]-[$([c;$(c(:c):c)])] |
| 37 | [$([S;D2](-[#0,#6]))]-[$([C;$(C(-;@[C,N,O,S])-;@[N,O,S])])] |
| 38 | [$([S;D2](-[#0,#6]))]-[$([c;$(c(:[c,n,o,s]):[n,o,s])])] |
| 39 | [$([S;D2](-[#0,#6]))]-[$([C;$(C(-;@C)-;@C)])] |
| 40 | [$([S;D2](-[#0,#6]))]-[$([c;$(c(:c):c)])] |
| 41 | [$([C;$(C(-;@[C,N,O,S])-;@[N,O,S])])]-[$([c;$(c(:[c,n,o,s]):[n,o,s])])] |
| 42 | [$([C;$(C(-;@[C,N,O,S])-;@[N,O,S])])]-;!@[$([C;$(C(-;@C)-;@C)])] |
| 43 | [$([C;$(C(-;@[C,N,O,S])-;@[N,O,S])])]-[$([c;$(c(:c):c)])] |
| 44 | [$([C;R1;$(C(-;@[C,N,O,S;R2])-;@[N,O,S;R2])])]-;@[$([C;R1;$(C(-;@[C;R2])-;@[C;R2])])] |
| 45 | [$([c;$(c(:[c,n,o,s]):[n,o,s])])]-[$([c;$(c(:[c,n,o,s]):[n,o,s])])] |
| 46 | [$([c;$(c(:[c,n,o,s]):[n,o,s])])]-[$([C;$(C(-;@C)-;@C)])] |
| 47 | [$([c;$(c(:[c,n,o,s]):[n,o,s])])]-[$([c;$(c(:c):c)])] |
| 48 | [$([C;$(C(-;@C)-;@C)])]-[$([c;$(c(:c):c)])] |
| 49 | [$([c;$(c(:c):c)])]-[$([c;$(c(:c):c)])] |


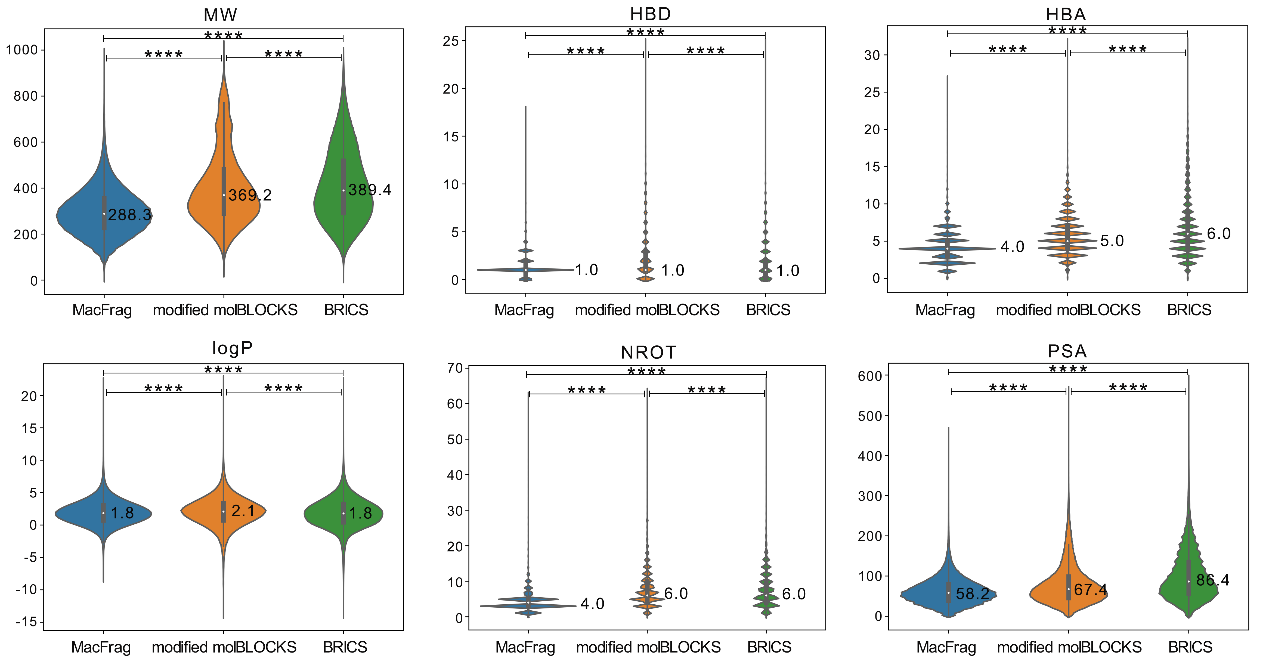


**Fig. S1.** Property values of fragments generated by MacFrag (maxBlocks =6), modified molBLOCKS (k =6), and BRICS implemented in RDKit (keepNonLeafNoldes =True), repsectively. The properties relevant to the RO3 include molecular weight （MW, <300）, the number of hydrogen bond donors (NHD, ≤3), the number of hydrogen bond acceptors (NHA, ≤3), logP ( ≤3), the number of rotatable bonds (NROT, ≤3), and polar surface area (PSA, ≤60). The two-sided Wilcoxon rank-sum test was used to calculate the statistical difference of each property and p value depicted by ****p < 0.0001. The median values are labeled to further display the discrepancies between the groups.


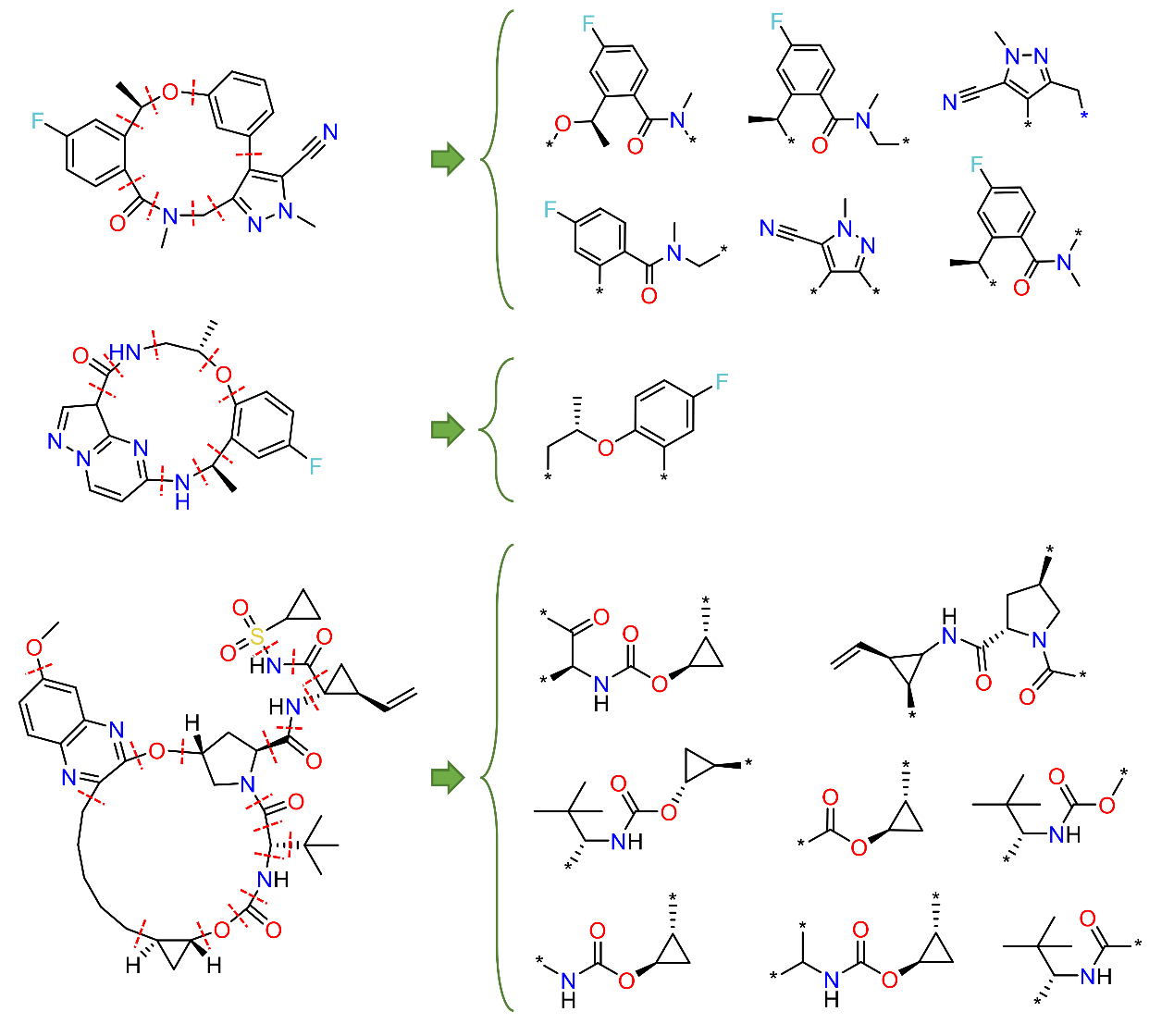


**Fig. S2.** Distinctive RO3-compliant fragments generated by MacFrag by breaking cyclic bonds. All breaking bonds identified by MacFrag are labeled with red dash lines. These fragments couldn’t be acquired by either BRICS or modified molBLOCKS through segmenting ChEMBL compounds with molecular weights lower than 1000.
